# Phytic acid-based nanomedicine against mTOR represses lipogenesis and immune response for metabolic dysfunction-associated steatohepatitis therapy

**DOI:** 10.1093/lifemeta/loae026

**Published:** 2024-06-18

**Authors:** Fenghua Xu, Shoujie Zhao, Yejing Zhu, Jun Zhu, Lingyang Kong, Huichen Li, Shouzheng Ma, Bo Wang, Yongquan Qu, Zhimin Tian, Junlong Zhao, Lei Liu

**Affiliations:** State Key Laboratory of Holistic Integrative Management of Gastrointestinal Cancers and Xijing Hospital of Digestive Diseases, Air Force Medical University, Xi’an, Shaanxi 710032, China; Key Laboratory of Modern Teaching Technology, Ministry of Education, Shaanxi Normal University, Xi’an, Shaanxi 710062, China; Department of General Surgery, Tangdu Hospital, Air Force Medical University, Xi’an, Shaanxi 710038, China; State Key Laboratory of Holistic Integrative Management of Gastrointestinal Cancers and Xijing Hospital of Digestive Diseases, Air Force Medical University, Xi’an, Shaanxi 710032, China; State Key Laboratory of Holistic Integrative Management of Gastrointestinal Cancers and Xijing Hospital of Digestive Diseases, Air Force Medical University, Xi’an, Shaanxi 710032, China; Key Laboratory of Modern Teaching Technology, Ministry of Education, Shaanxi Normal University, Xi’an, Shaanxi 710062, China; State Key Laboratory of Holistic Integrative Management of Gastrointestinal Cancers and Xijing Hospital of Digestive Diseases, Air Force Medical University, Xi’an, Shaanxi 710032, China; Department of Thoracic Surgery, Tangdu Hospital, Air Force Medical University, Xi’an, Shaanxi 710038, China; Department of General Surgery, Tangdu Hospital, Air Force Medical University, Xi’an, Shaanxi 710038, China; Key Laboratory of Special Functional and Smart Polymer Materials of Ministry of Industry and Information Technology, School of Chemistry and Chemical Engineering, Northwestern Polytechnical University, Xi’an, Shaanxi 710072, China; Key Laboratory of Special Functional and Smart Polymer Materials of Ministry of Industry and Information Technology, School of Chemistry and Chemical Engineering, Northwestern Polytechnical University, Xi’an, Shaanxi 710072, China; State Key Laboratory of Holistic Integrative Management of Gastrointestinal Cancers, Department of Medical Genetics and Developmental Biology, Air Force Medical University, Xi’an, Shaanxi 710032, China; Department of Pediatrics, Tangdu Hospital, Air Force Medical University, Xi’an, Shaanxi 710000, China; State Key Laboratory of Holistic Integrative Management of Gastrointestinal Cancers and Xijing Hospital of Digestive Diseases, Air Force Medical University, Xi’an, Shaanxi 710032, China

**Keywords:** MASH, CePA, mTOR, inflammatory response, lipid metabolism

## Abstract

Metabolic dysfunction-associated steatohepatitis (MASH) is one of the most common chronic liver diseases and is mainly caused by metabolic disorders and systemic inflammatory responses. Recent studies have indicated that the activation of the mammalian (or mechanistic) target of rapamycin (mTOR) signaling participates in MASH progression by facilitating lipogenesis and regulating the immune microenvironment. Although several molecular medicines have been demonstrated to inhibit the phosphorylation or activation of mTOR, their poor specificity and side effects limit their clinical application in MASH treatment. Phytic acid (PA), as an endogenous and natural antioxidant in the liver, presents significant anti-inflammatory and lipid metabolism-inhibiting functions to alleviate MASH. In this study, considering the unique phosphate-rich structure of PA, we developed a cerium-PA (CePA) nanocomplex by combining PA with cerium ions possessing phosphodiesterase activity. CePA intervened in the S2448 phosphorylation of mTOR through the occupation effect of phosphate groups, thereby inhibiting the inflammatory response and mTOR-sterol regulatory element-binding protein 1 (SREBP1) regulation axis. The *in vivo* experiments suggested that CePA alleviated MASH progression and fat accumulation in high-fat diet-fed mice. Mechanistic studies validated that CePA exerts a liver-targeted mTOR repressive function, making it a promising candidate for MASH and other mTOR-related disease treatments.

## Graphical Abstract

**Graphical Abstract:**
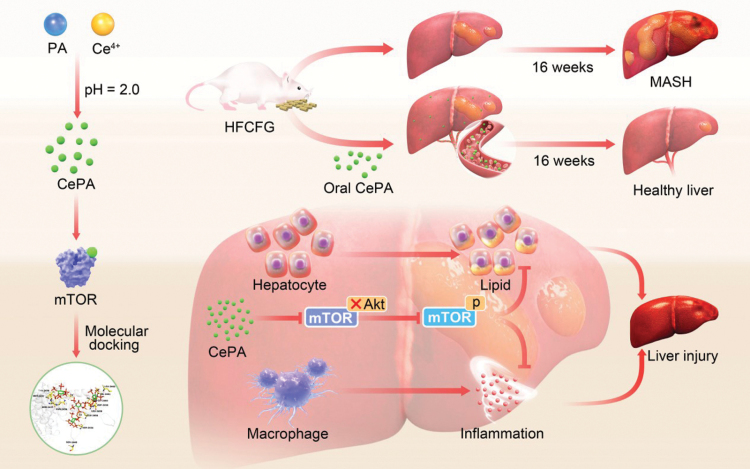

## Introduction

Metabolic dysfunction-associated steatohepatitis (MASH), one of the most common chronic liver diseases, has become a major contributor to cirrhosis and liver cancer, resulting in a large number of deaths on a global scale [1–3]. Although the incidence of MASH has gradually increased, the absence of effective medicine or approaches limits therapy for MASH [4]. An increasing number of studies suggest that inflammation and lipid metabolism disorders act as the main factors and characteristics of MASH [5]. As an important signaling molecule regulating lipid metabolism and inflammatory response, the mammalian (or mechanistic) target of rapamycin (mTOR) could play an important role in MASH progression. The function of mTOR depends on the phosphorylation of its S2448 amino acid residue. It has been shown that mTOR expression levels are upregulated after high-fat/calorie diet plus high fructose/glucose in drinking water (HFCD-HF/G, or HFCFG) feeding [6] and that administration of inhibitors of mTOR inhibits hepatic steatosis and influences the pathology of metabolic dysfunction-associated fatty liver disease (MAFLD) by modulating the inflammatory response [7, 8]. However, molecular inhibitors targeting mTOR present limitations such as poor specificity, low targeting, and weak tissue affinity. Therefore, the development of MASH therapeutics targeting mTOR is crucial.

A large body of evidence has suggested that disorders in the lipid metabolism pathway contribute to the initiation and progression of MASH [9–11]. The disturbance between anabolism and catabolism is the main reason for the liver lipid metabolism imbalance. During physiological development, hepatocyte-derived molecules participate in liver metabolism regulation. Phytic acid (inositol-1,2,3,4,5,6-hexakisphosphate, PA), an endogenously synthesized metabolite in the liver, can alleviate hepatic inflammation and attenuate the pathological phenotype of MASH [12, 13]. Further mechanistic studies indicated that PA has a profound impact on inhibiting lipid anabolism and suppressing proinflammatory cytokine production through mTOR signaling modulation [14]. However, PA can be converted into diphosphoinositol pentakisphosphate (PP-IP_5_ or IP_7_), a inositol pyrophosphate, by a variety of enzymes *in vivo*, thus losing its anti-inflammatory therapeutic activity. In particular, the hepatic microenvironment during MASH significantly decreases PA production and secretion, exacerbating hepatic lipid damage [15, 16]. Therefore, exogenous PA supplementation shows better prospects for MASH treatment. However, the indefinite targeting and instability *in vivo* lead to weak drug utilization in the liver [17], which limits its clinical translation and application.

In recent years, nanomedicine has shown promising application prospects in the treatment of liver diseases. In particular, cerium-based nanomaterials possess significant antioxidant and anti-inflammatory effects as well as liver tissue targeting and specificity [18–30]. The present studies have shown that metal complexes of PA can be better taken up by tissues and cells [31]. Cerium-based nanoenzymes possess phosphatase activity and can modulate the phosphorylation levels of a variety of proteins. It also has a strong anti-inflammatory effect. The current design of small molecule inhibitors mainly focuses on the occupancy effect and competitive antagonism to the active domain of the target protein. Considering that PA is enriched with phosphate groups, we designed cerium-PA coordination nanomedicine (CePA) with the expectation that they could be used for targeting the inhibition of mTOR phosphorylation. In the current study, CePA was verified to spatially block the phosphorylation and activation of mTOR to alleviate hepatic steatosis, lipid-associated damage, and fibrosis in MASH *in vitro* and *in vivo*.

## Results and discussion

### The synthesis, characteristics, and mTOR repression of CePA

**Figure 1:**
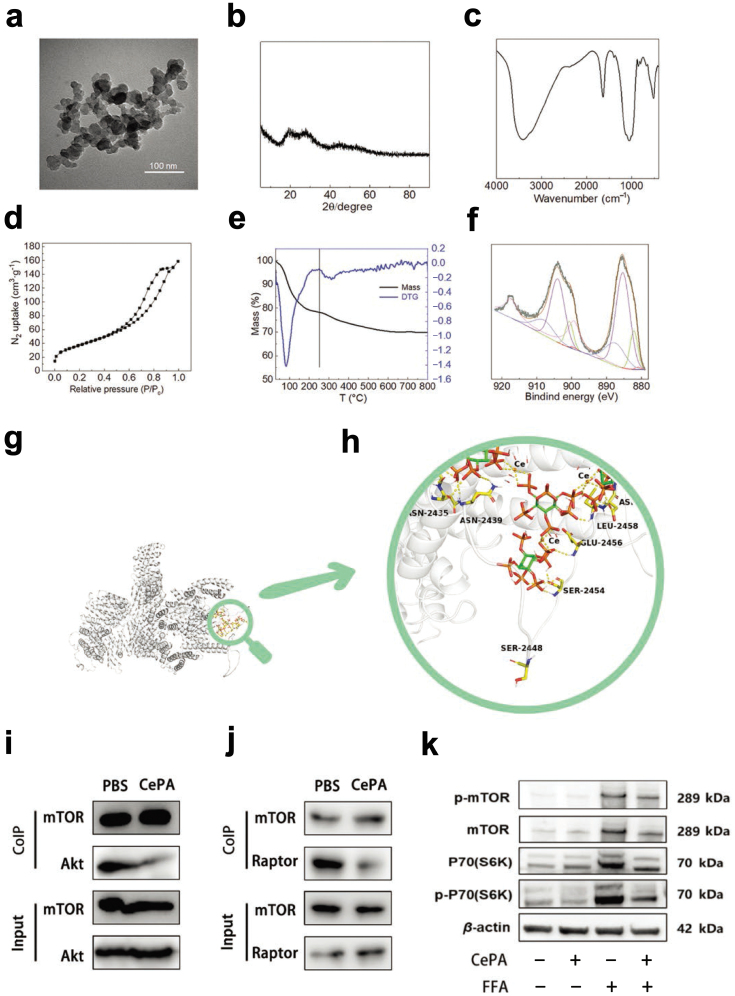
Synthesis and characteristics of CePA. (a) Transmission electron microscopy of CePA. (b and c) X-ray diffraction and infrared spectroscopy analysis of nanomaterials to verify the formation of CePA particles. (d) Specific surface area analysis of CePA. (e) Thermogravimetric curves of CePA. (f) XPS of CePA. (g and h) Calculated molecular structure of CePA. (i and j) CoIP to detect the interaction of CePA and Akt, as well as CePA and Raptor in hepatocytes treated with FFA. (k) Western blot analysis of the expression of p-mTOR and p-70(S6K) in cells treated with or without CePA after FFA stimulation for 24 h. *n* = 3.

Phosphatidylinositol can bind to a variety of proteins in addition to its signaling regulatory function [32]. Taking advantage of the potential protein binding function of PA, we designed and synthesized CePA. The morphology of CePA was observed by transmission electron microscopy, and the size of CePA was 30−50 nm (Fig. 1a). The formation of CePA particles was verified by X-ray diffraction and Fourier transform infrared spectroscopy (Fig. 1b and c). The specific surface area analysis of the material showed that CePA had a large number of pores, which was beneficial for subsequent biological applications (Fig. 1d). The PA content in the CePA was 27.6 wt % according to the thermogravimetry result (Fig. 1e). X-ray photoelectron spectroscopy (XPS) of cerium in CePA showed that the chemical valence of cerium in CePA was a mixed state of trivalence and tetravalence, and the percentage of Ce^3+^ was 51.4%. The relatively high ratio of Ce^3+^ can enhance the antioxidant activity and further be used to treat liver oxidative injury (Fig. 1f). Since PA is mostly degraded in the gastrointestinal tract, scanning electron microscopy was used to observe the structural changes of CePA in gastric juice and intestinal juice after 3, 6, 9, and 12 h of digestion, and the results showed that CePA was structurally stable in the gastrointestinal tract (Supplementary Fig. S1). Besides, we also compared PA and CePA in AML12 cells treated with free fatty acid (FFA). It was found that CePA was more able to stabilize cell activity (Supplementary Fig. S2a and b). At the same time, CePA had more stable and effective lipid protection on liver than PA (Supplementary Fig. S2c).

Next, we analyzed the physical interaction of CePA and mTOR and determined its interventional effects. Some studies have shown that the mTOR signaling pathway directly regulates MASH by activating lipogenesis via multiple mechanisms, including sterol regulatory element-binding proteins (SREBPs), acetyl-CoA carboxylase (ACC), fatty acid synthase (FASN), and ATP citrate lyase (ACLY) [33]. Phosphorylation of the S2448 and p70(S6K) residue is crucial for mTOR activation. Under the stress of environmental stimulation, mTOR is bound to the upstream kinase Akt (protein kinase B), resulting in S2448 phosphorylation and promoting the formation of the mTOR complex 1 (MTOC1) with Raptor [34]. The above evidence indicated that mTOR signaling is needed for MASH progression. Calculation of the molecular structure of CePA and molecular docking results of CePA with the mTOR protein showed that CePA could bind to mTOR and was close to the serine residue at S2448 in the spatial conformation (Fig. 1g and h), which indicated that CePA might influence mTOR S2448 phosphorylation by Akt topologically. Therefore, we carried out a coimmunoprecipitation (CoIP) assay to verify the interaction between mTOR and Akt after CePA treatment. The results suggested that CePA treatment significantly repressed the binding of mTOR with Akt as well as further Raptor recruitment (Fig. 1i and j). Furthermore, we investigated the influence of CePA on mTOR phosphorylation and activation. The examination results showed that FFA promoted the phosphorylation of mTOR and p70(S6K) in the hepatocyte cell line AML12, which could be retrieved by CePA treatment (Fig. 1k). These results indicated that the synthesized CePA presents effective mTOR repression ability through both theoretical docking and experimental validation, which might play an important role in MASH therapy.

### CePA attenuates lipid accumulation and inflammation by regulating mTOR signaling

Previous studies have demonstrated that increased mTOR facilitates lipid synthesis by upregulating the expression of related enzymes, such as ACC and FASN [35]. Furthermore, the immune regulation function of mTOR signaling could evoke liver macrophage activation to facilitate further inflammatory responses, cell apoptosis, and liver damage, which finally induce hepatocyte steatosis [36]. Considering that CePA could bind with mTOR and inhibit mTOR activation and the MTOC1 complex (Fig. 1), we investigated the effect of CePA on mTOR-mediated lipid aggregation and metabolism disorders in hepatocytes.

**Figure 2:**
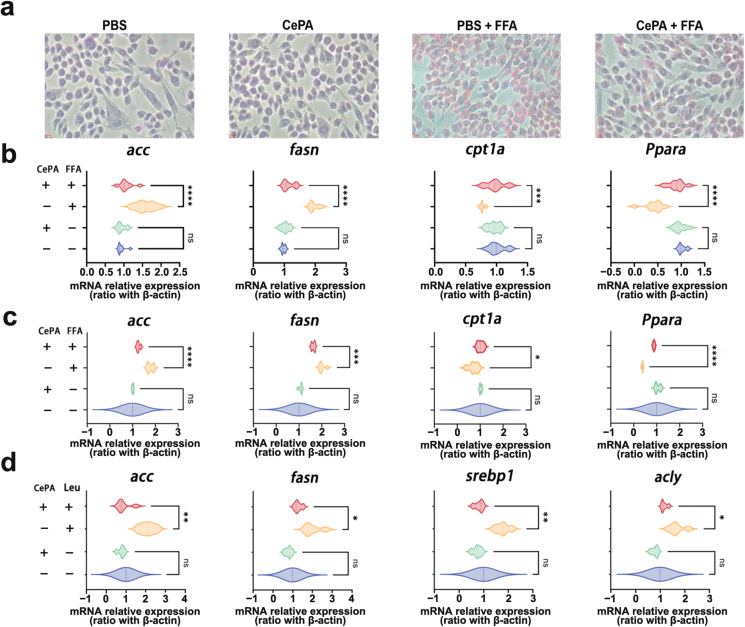
CePA regulates lipid aggregation in human primary hepatocytes and AML12 cells. (a) Oil Red O staining of the NH cell lines of mice (AML12) treated with PBS and CePA with or without FFA stimulation for 24 h. Scale bar, 100 μm. (b and c) RT-PCR detection of mRNA levels for lipid metabolism-related genes (*acc*, *fasn*, *cpt1a*, and *Ppara*) in human primary hepatocytes (b) and AML12 (c) treated with or without CePA after FFA stimulation for 24 h. *n* = 3. (d) RT-PCR detection of mRNA levels for lipid metabolism-related genes (*acc*, *fasn*, *srebp1*, and *acly*) in hepatocytes treated with or without CePA after adding leucine or PBS. Leucine, *n* = 3.

**Figure 3:**
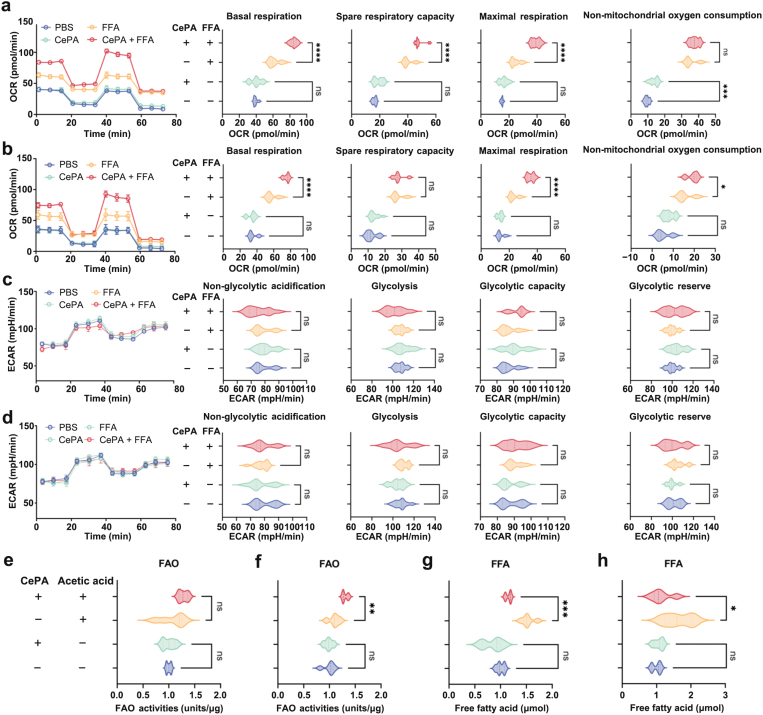
(a and b) OCR measurements. A total of 1 mmol/L oligomycin, 3 mmol/L FCCP, and 0.5 mmol/L antimycin with rotenone were injected sequentially to the sample plate at the time points indicated. OCR values are presented as a bar graph in human primary hepatocytes (a) and AML12 (b) treated with or without CePA after FFA stimulation for 24 h. *n* = 3. (c and d) ECAR measurements. A total of 10 mmol/L glucose, 1 mmol/L oligomycin, and 50 mmol/L 2-DG were injected sequentially to the sample plate at the time points indicated. ECAR values are presented as a bar graph in human primary hepatocytes (c) and AML12 (d) treated with or without CePA after FFA stimulation for 24 h. *n* = 3. (e and f) Homogenates were prepared from human primary hepatocytes (e) and AML12 (f) treated with or without CePA after acetic acid stimulation for 24 h. FAO activities were assayed by the FAO Assay kit. *n* = 3. (g and h) Plasma FFA levels measured using ab65341 in human primary hepatocytes (g) and AML12 (h) treated with or without CePA after acetic acid stimulation for 24 h. *n* = 3. The data are expressed as the mean ± SEM. ^*^P < 0.05, ^**^P < 0.01, ^***^P < 0.001 vs. PBS.

Using an Oil Red O staining assay, we detected the intracellular lipid content in normal hepatocyte (NH) cell line AML12 after treatment with PBS, Ce, PA, and CePA for 24 h in the presence of FFA stimulation to evaluate whether CePA can reduce lipid accumulation [37, 38]. Fig. 2a demonstrates the intense red staining in AML12 cells upon oleic acid (OA) administration, whereas cotreatment with CePA decreased the intensity of the color comparatively. These results revealed that CePA could alleviate FFA-induced lipid droplet aggregation in hepatocytes, which indicated that CePA inhibited the overgeneration of lipids (Fig. 2a). To identify the mechanisms involved in the reduction in hepatic lipid accumulation by CePA, we investigated the expression of lipogenesis. A recent study demonstrated that FFA promotes the protein expression levels of SREBP1, ACC, and FASN [39]. Reverse transcription-polymerase chain reaction (RT-PCR) was used to examine the mRNA expression levels of lipid metabolism-related genes in human primary hepatocytes and mouse NH cell lines (AML12), and the results showed that CePA could also significantly alleviate the upregulation of lipid metabolism-related gene (*acc* and *fasn*) expression and downregulation of lipid synthesis-related gene (carnitine palmitoyl transferase 1a (*cpt1a*) and peroxisome proliferator-activated receptor alpha (*Ppara*)) expression caused by FFA stimulation (Fig. 2b and c). The above results suggested that CePA could improve FFA-induced disorders of lipid metabolism. To further confirm that CePA plays a regulatory role by inhibiting mTOR, we added an mTOR agonist (leucine) for further analysis (Fig. 2d). Branched-chain amino acids such as leucine potentially facilitate mTOR activation in an Akt-independent manner. Leucine has been found to activate mTORC1 via inhibition of tuberous sclerosis complex 1 (TSC1)/TSC2 [40]. To determine functional changes in cell metabolic activity, we assessed the oxygen consumption rate (OCR), a measure of overall mitochondrial respiration, of human primary hepatocytes and AML12 cells (Fig. 3a and b). Our data showed that basic respiration significantly increased after the addition of CePA and reserve respiration significantly increased after the addition of rotenone. The increase in oxygen consumption after the addition of uncoupling agent carbonylcyanide *p*-trifluoromethoxyphenylhydrazone (FCCP) reflected the mitochondrial reserve function. We found that the maximal respiration significantly increased after the addition of FCCP. Our results indicated that CePA facilitated the oxidative phosphorylation of FFA. To further determine the functional changes in cellular metabolic activity, we evaluated the extracellular acidification rate (ECAR) of human primary hepatocytes and AML12 cells (Fig. 3c and d), the results showing no significant changes in different treatments. We further used the fatty acid oxidation (FAO) assay to detect FAO after CePA treatment in the presence of FFA (Fig. 3e and f). The results were consistent with the seahorse detection that CePA promoted FAO in human primary hepatocytes. In addition, the Free Fatty Acid Assay Kit was used to detect the synthesis of FAs with the stimulation of acetic acid after treatment with CePA (Fig. 3g and h). We found that CePA could inhibit the synthesis of FAs to a certain extent in human primary hepatocytes and AML12 cells. The above results indicated that CePA could repress lipid metabolism in hepatocytes by blocking phosphorylation and repressing the mTOR signaling pathway.

**Figure 4:**
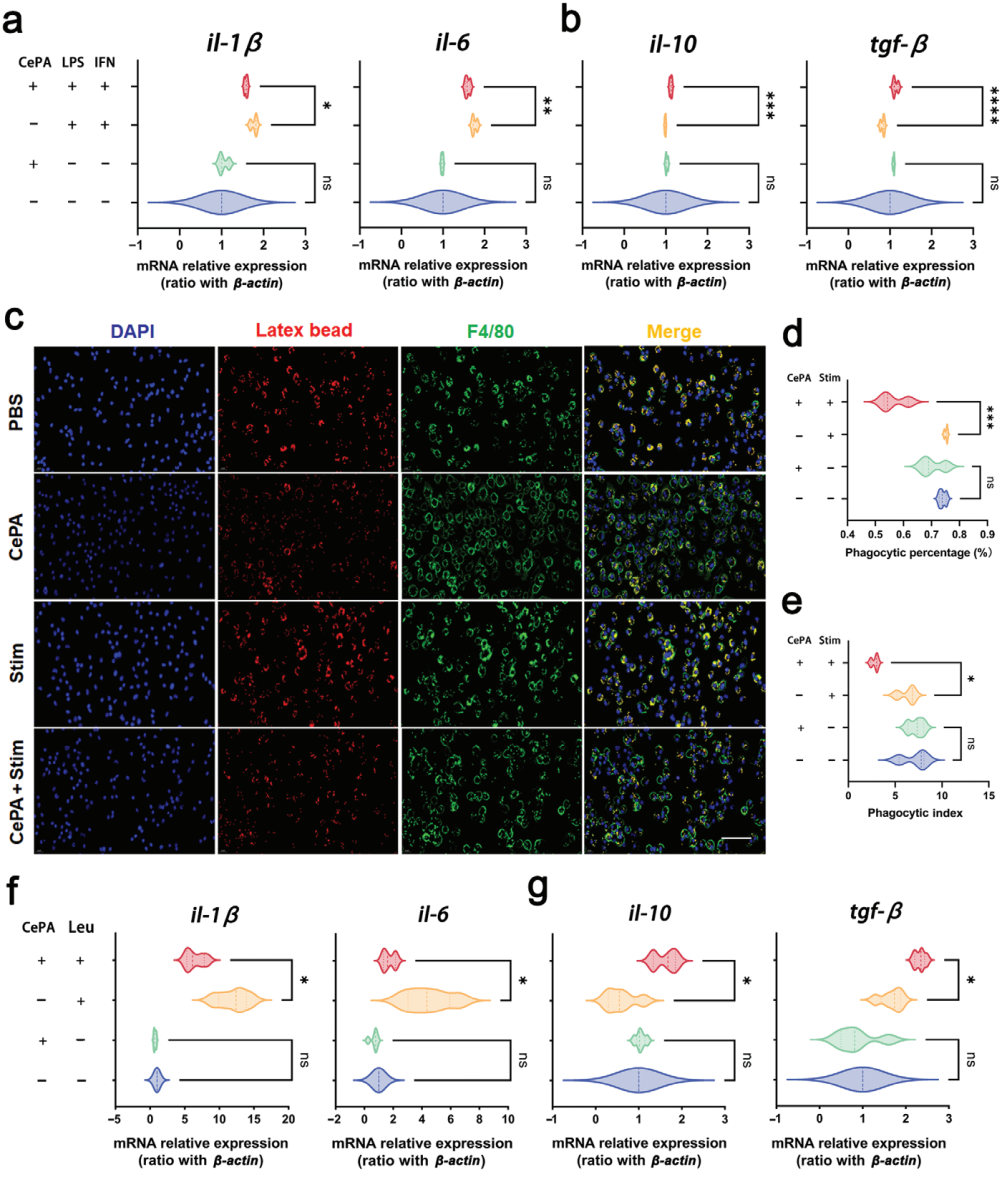
CePA regulates inflammation *in vitro*. (a and b) RT-PCR results of the mRNA levels of pro-inflammatory (*il-1β* and *il-6*) (a) and anti-inflammatory genes (*il-10* and *tgf-β*) (b) from BMDMs treated with PBS, LPS, IFN-γ, and CePA for 24 h. *n* = 5. (c) Phagocytosis of macrophages is evaluated by immunofluorescence assay. Blue represents 4ʹ,6-diamidino-2-phenylindole (DAPI) for the nucleus; green represents F4/80; red represents latex beads. Scale bar, 100 μm. (d and e) The statistical chart. (f and g) mRNA levels of inflammatory response gens (*il-1β, il-6, il-10,* and *tgf-β*) in hepatocytes treated with or without CePA after the addition of leucine or PBS. Leucine, *n* = 3. The data are expressed as the mean ± SEM. ^*^P < 0.05, ^**^P < 0.01, ^***^P < 0.001 vs. PBS.

The liver is rich in a large number of innate immune cells such as hepatic macrophages. Studies have shown that innate immune cells and related molecules participate in the pathogenesis of various liver diseases. Hepatic macrophage activation and infiltration have been observed in the development of MASH. To further investigate the potential importance of the regulated macrophage inflammatory response as a mechanism by which CePA protected against the pathogenesis of MASH, the mRNA expression levels of cytokine markers in macrophages were measured. Lipopolysaccharide (LPS) and interferon-gamma (IFN-γ) were used to stimulate macrophage M1 polarization, and CePA was added for therapy. The RT-PCR results suggested that compared with the PBS group, CePA could significantly reduce the expression of pro-inflammatory factors (interleukin-1 (*il-1* and *il-6*) (Fig. 4a), while increasing the expression of anti-inflammatory factors (*il-10* and transforming growth factor-β (*tgf-β*)) (Fig. 4b) after IFN-γ and LPS treatment. These results indicated that CePA can significantly ameliorate the inflammatory response of macrophages.

Macrophages are first responder cells in the innate immune response, and their phagocytic ability is an important part of the innate immune system and is critical for homeostasis of the host. Impairment in phagocytosis has been associated with numerous diseases and disorders, including MASH. Different cytokines have been shown to affect the phagocytic process, and many cytokines, such as TNFα, LPS, and IFN-γ, have been shown to promote the phagocytic function of macrophages, which mainly play a role in promoting the development of inflammation and phagocytosis. Therefore, we examined the phagocytic function of the activation of M1-type macrophages (classically activated macrophages) by LPS and IFN-γ, and the immunofluorescence results showed that CePA could effectively inhibit the phagocytosis of macrophages labeled with F4/80 (Fig. 4c). Similarly, CePA significantly increased the phagocytic percentage and phagocytic index compared with the stim group (Fig. 4d and e). In addition to lipid metabolism, mTOR signaling regulates the inflammatory response and immune cell development [41]. Many studies have demonstrated that the activation of mTOR and its downstream MTOC1 complex promotes macrophage M1 polarization and cytokine secretion [42, 43]. Similar to the above results, additional mTOR agonist leucine was added to the culture medium of macrophages to activate the mTOR signaling pathway, which in turn induced an inflammatory response. The RT-PCR results suggested that, compared with the PBS group, CePA could significantly reduce the expression of pro-inflammatory factors (*il-1* and* il-6*) (Fig. 4f) while increasing the expression of anti-inflammatory factors (*il-10* and *tgf-β*) (Fig. 4g) after treatment with mTOR agonist leucine. These results indicated that CePA could improve macrophage polarization by inhibiting mTOR activation.

### CePA depresses mTOR-mediated lipid metabolism disorders during MASH progression

**Figure 5:**
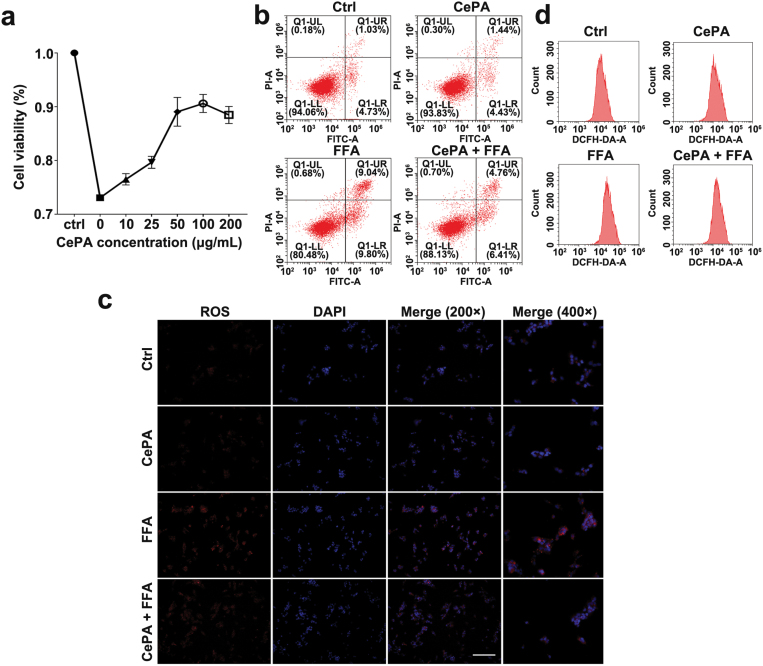
The safety of CePA materials. (a) Cell viability after CePA treatment. (b) Flow cytometry showed that CePA reduces oxidative stress stimulated by FFAs. (c) Flow cytometry showed apoptosis in the Ctrl, CePA, FFA, and CePA + FFA groups. Scale bar, 100 μm. (d) Immunofluorescence showed that CePA reduces oxidative stress stimulated by FFAs. Magnification: 200× and 400×.

Our results suggest that CePA alleviates FFA-induced lipid metabolism disorder and lipid accumulation in hepatocytes. To verify whether CePA itself was safe for mice, we tested the inflammatory infiltration of the lung, liver, kidney, heart, and spleen of CePA-fed and PBS-fed mice (Supplementary Fig. S3a) and the cell viability of AML12 and Mφ after CePA treatment (Supplementary Fig. S3b and c). The results showed that CePA had certain biosafety properties. To determine the cytotoxicity and optimal concentration of CePA to hepatocytes, AML12 cells were exposed to various concentrations (10, 25, 50, 100, and 200 µg/mL) of CePA for 24 h. Cholecystokinin octapeptide (CCK8) measurements showed that the cells had good cell viability under normal culture conditions and were significantly reduced with FFA treatment, but CePA significantly improved cell viability in a dose-dependent manner. CePA was able to neutralize FFA-induced toxicity in a dose-dependent manner (Fig. 5a). AML12 cells were treated with FFA for 24 h in the presence or absence of CePA. Quantitative analysis of apoptotic cells showed that FFA induced rapid apoptosis after exposure for 48 h, whereas CePA strongly saved the cells (Fig. 5b). There is ample evidence that the production of too much O_2_ by FAs can lead to lipotoxicity. High levels of saturated FAs also stimulate the plasma membrane to produce more reactive oxygen species (ROS) by NADPH oxidase (NOX). In this study, a large amount of total ROS production was detected in FFA-treated cells, and this effect was significantly inhibited by the addition of CePA (Fig. 5a and d). These findings suggested that CePA alone does not affect apoptosis or oxidative stress and has a good biosafety profile. CePA inhibits oxidative stress caused by FFA overload, which may contribute to liver cell survival [44].

**Figure 6:**
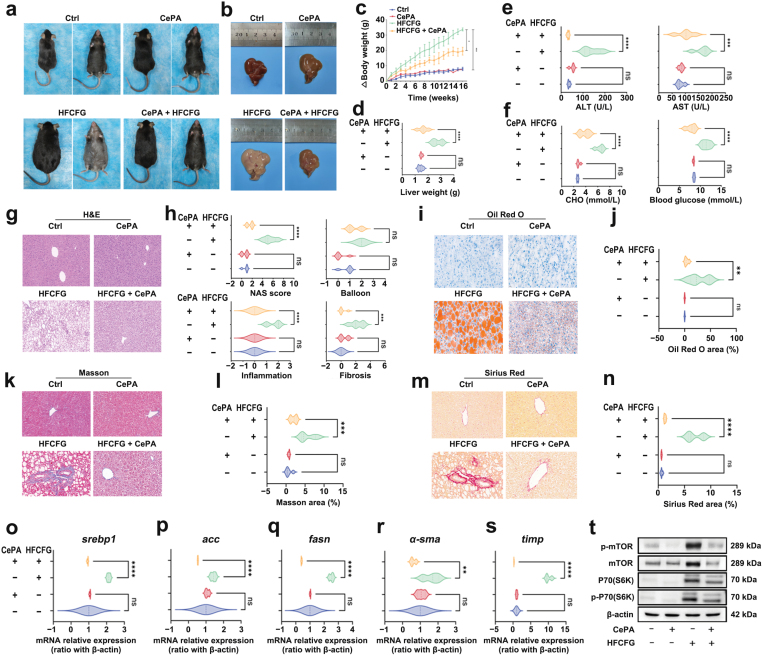
CePA regulates metabolic disorders *in vivo*. (a and b) Photographs of mice and mouse livers fed a normal or HFCFG diet with or without CePA for 16 weeks. Scale bar, 1 cm. (c and d) Body weight growth curve (c) and liver weight (d) of mice in the indicated groups. *n* = 3. (e) Enzyme-linked immunosorbent assay (ELISA) results of serum concentrations of ALT (left) and AST (right) in mice fed a normal or HFCFG diet with or without CePA. (f) Total cholesterol concentrations and blood glucose of mice in the indicated groups. (g) H&E staining of mice in the Ctrl, CePA, HFCFG, and HFCFG + CePA groups. Scale bar, 100 µm. (h) NAS of liver sections and quantitative analysis results of balloon, inflammation, and fibrosis levels of mouse liver sections in the indicated groups. (i) Oil Red O staining images in Ctrl, CePA, HFCFG, and HFCFG + CePA mice. (j) Oil Red O area in the indicated groups. (k) Masson staining of mice in Ctrl, CePA, HFCFG, and HFCFG + CePA groups. Scale bar, 100 µm. (l) Masson area in the indicated groups. (m) Sirius Red staining of mice in Ctrl, CePA, HFCFG, and HFCFG + CePA groups. Scale bar, 100 µm. (n) Sirius Red area in the indicated groups. (o–q) Relative mRNA expression of lipid metabolism-related genes *srebp1* (o), *acc* (p), and *fasn* (q) in the indicated groups. (r and s) mRNA levels of fibrosis-related markers *α-sma* and *timp* in the indicated groups. (t) Western blot to detect the expression of p-mTOR and p-P70(S6K) after HFCFG feeding. Scale bar, 100 μm. The data are expressed as the mean ± SEM. ^*^P < 0.05, ^**^P < 0.01, ^***^P < 0.001 for HFCFG vs. Ctrl or HFCFG + CePA vs. HFCFG; ns indicates not statistically significant.

Our findings provided evidence that CePA can regulate lipid metabolism and inflammation *in vitro*. Next, we mainly focused on the regulation of CePA *in vivo*. MASH is a metabolic disease associated with obesity, and an increasing number of studies have shown that it will cause significant hepatic steatosis in HFCFG-fed mice [45]. Therefore, C57BL/6 mice were fed an HFCFG diet for 16 weeks to establish MASH models and fed an HFCFG diet supplemented with 1% CePA to evaluate the effect of CePA. On the basis of liver targeting and biosafety, we further analyzed the therapeutic effect of CePA on MASH. By observing the appearance, with increased HFCFG feeding time, the body weight of mice in the Ctrl and CePA groups increased slowly after three weeks, with no significant sexual differences between the two groups. However, the weight of mice in the HFCFG group showed a significant increase, with an average weight gain of 32–36 g, while the HFCFG + CePA group was able to reduce the weight induced by a high-fat diet by an average of 10–15 g at 16 weeks. CePA administration significantly reversed the HFCFG-induced phenomenon (Fig. 6a and c). Macroscopic observation showed that the livers of HFCFG-fed mice were enlarged and yellow with white spots on the liver surface. In parallel, CePA can effectively alleviate HFCFG-induced body weight increases. Consistent with body weight, the increased liver weight was also restored by CePA (Fig. 6b and d). Generally, the development of MASH is accompanied by metabolic syndrome, including high blood glucose, liver metabolism disorder, and liver injury [46, 47]. Therefore, the concentrations of alanine transaminase (ALT), aspartate transaminase (AST), and cholesterol (CHO) were measured in the serum of mice subjected to different treatments. The results suggested that the levels of ALT, AST, and CHO in the CePA groups were significantly lower than those in the HFCFG group. Meanwhile, the blood glucose levels were also decreased after CePA treatment (Fig. 6e and f). These results indicated that HFCFG-fed mice showed significant metabolic disorders, and the administration of CePA improved these phenomena, suggesting that CePA may be more effective in the treatment of metabolic liver disease and metabolic syndrome.

MASH is characterized by hepatic steatosis, hepatocyte damage (ballooning degeneration), inflammation, and steatosis [1, 48]. We mainly focused on the protective effect of CePA on MASH by histological staining. Hematoxylin and eosin (H&E) staining was used to observe liver tissue injury in mice. By observing the appearance, with increased HFCFG feeding time on HFCFG, mice in the Ctrl and CePA groups increased slowly after three weeks, with no significant sexual differences between the two groups. However, the weight of mice in the HFCFG group showed a significant increase, with an average weight gain of 32–34 g, while the HFCFG + CePA group was able to reduce the weight induced by a high-fat diet by an average of 12–14 g at 16 weeks. The results showed that HFCFG diet could increase the MASH activity score (NAS) and elicit obvious ballooning degeneration in liver tissue, hepatic interlobular inflammatory infiltration, and steatosis, suggesting that HFCFG could significantly aggravate liver injury, and CePA could significantly reverse the above phenomenon (Fig. 6g and h). The results of Oil Red O staining indicated that CePA could effectively inhibit the significant accumulation of lipid droplets in the liver tissue of mice induced by HFCFG diet (Fig. 6i and j). These results showed that HFCFG-fed mice had significant steatosis and lipid accumulation in liver tissue, and the administration of CePA significantly improved the above phenomenon. The outcomes of MASH and liver-specific diseases are closely related to the degree of liver fibrosis. Approximately 40% of MASH patients will have fibrosis progression. Although liver fibrosis is not clinically a diagnostic criterion for MASH, the degree of liver fibrosis can predict the outcome of MASH, including the mortality of MASH patients [49–51]. Therefore, we detected the degree of HFCFG-induced liver tissue fibrosis by Masson (Fig. 6k and l) and Sirius Red (Fig. 6m and n) staining, and we found that CePA could effectively improve the liver tissue fibrosis induced by HFCFG [52]. These data suggested that CePA plays an important regulatory role in diet-induced murine models of MASH. Then, we examined the expression of genes related to lipid synthesis (*srebp1, acc, and fasn*) in liver tissue, and the results showed that CePA could effectively reduce the HFCFG-induced elevated expression of lipid synthesis-related genes (Fig. 6o–q). It has been reported that mTOR regulates the expression of adipogenic enzymes through the regulation of SREBP1, a transcription factor that controls lipid synthesis [34]. These results suggest that CePA is involved in regulating hepatic steatosis and reducing hepatic fat deposition through targeting and regulating mTOR and thus affecting SREBP1 expression, which has a protective effect against high-fat diet-induced liver injury in mice. At the same time, CePA attenuated the HFCFG-induced increase in fibrosis-related marker gene (α-smooth muscle actin (*α-sma*) and tissue inhibitor of metalloproteinase (*timp*)) expression (Fig. 6r and s). Furthermore, we investigated the influence of CePA on mTOR and its p-P70(S6K) phosphorylation, suggesting that CePA reduced mTOR phosphorylation even in the livers of HFCFG-fed mice (Fig. 6t). These results suggested that CePA plays a regulatory role in MASH by inhibiting the phosphorylation of mTOR. Taken together, our findings suggest that CePA can protect HFCFG-induced MASH by regulating lipid droplet aggregation, fat deposition, and liver fibrosis in liver tissue.

### CePA ameliorates HFCFG-induced liver and systemic inflammation by inhibiting mTOR activation

**Figure 7:**
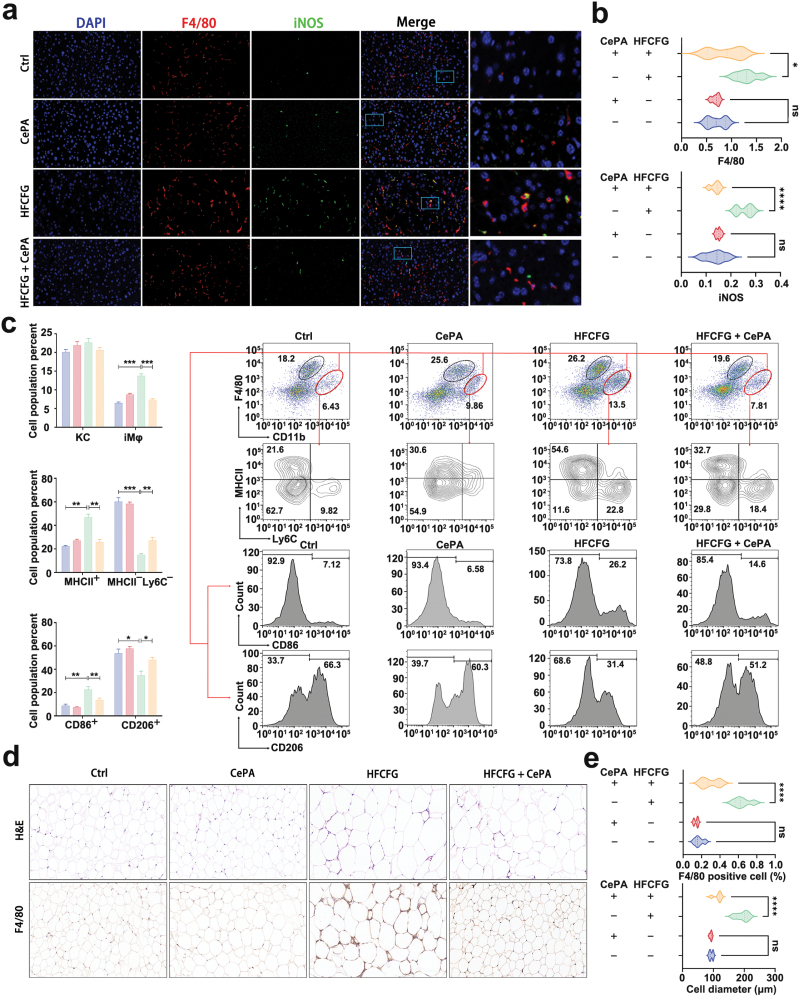
CePA regulates inflammation *in vivo*. (a) Immunofluorescence imaging of mice treated with or without CePA after HFCFG feeding. Blue represents DAPI for the nucleus; red represents F4/80; green represents iNOS. Magnification: 400×. (b) Statistical graph of the number of F4/80^+^ cells and iNOS^+^ cells. (c) Representative flow cytometry plots and statistical data showing the percentages of macrophages, MHC II^+^ cells, MHC II^−^Ly6C^−^ cells, CD86^+^ cells, and CD206^+^ cells in the indicated groups. (d) H&E and immunohistochemical staining to observe white adipose tissue in mice. (e) Statistical chart of (d) to present macrophage infiltration and white adipocyte diameter. Scale bar, 100 μm. The data are expressed as the mean ± SEM. ^*^P < 0.05, ^**^P < 0.01, ^***^P < 0.001 for HFCFG vs. Ctrl or HFCFG + CePA vs. HFCFG.

Lipotoxicity and inflammatory cytokines are important factors for hepatocellular damage and the exacerbation of MASH [53]. Studies and our previous *in vitro* data have shown that mTOR is involved in the regulation of the inflammatory response in MASH [7]. Therefore, we next detected the local immune response in the liver through fluorescence-activated cell sorting (FACS) and immunofluorescence staining. Hepatic macrophages, as the largest immune population in the liver, play a crucial role in the immune response. When MASH injury occurs in the liver, macrophages are recruited to the injury site and produce a series of cytokines and chemokines, which could further recruit other immune cells for adaptive responses [53, 54]. A recent study demonstrated that the expression of inducible nitric oxide synthase (iNOS) in liver macrophages is significantly increased in MASH model mice and MASH patients. iNOS-derived nitric oxide (NO) in HFCFG conditions plays an important role in the process of hepatic fibrosis and inflammation, and the deficiency of iNOS strongly accelerates progression to MASH. Therefore, we first examined the regulatory effect of CePA on macrophages in an HFCFG-induced MASH model. Therefore, we carried out co-staining of macrophages (F4/80) and iNOS in HFCFG-induced mouse liver tissue. Immunofluorescence results showed that CePA could significantly inhibit the recruitment and infiltration of HFCFG-induced macrophages and improve macrophage activation. In addition, CePA significantly reduced the expression of HFCFG-induced iNOS (Fig. 7a and b).

CD11b and F4/80 are considered as surface markers of macrophages [55, 56]. The CD11b^+^F4/80^+^ cells were gated and calculated by FACS analysis, indicating that HFCFG feeding significantly increased the number of macrophages and that the addition of CePA could reverse the above phenomena. Meanwhile, the expression of lymphocyte antigen 6 complex (Ly6C) and major histocompatibility complex class II (MHC II) was related to macrophage maturation. During macrophage differentiation, the level of Ly6C was decreased, and the expression of MHC II was upregulated. A previous study demonstrated that mature macrophages with a Ly6C^−^ MHC II^+^ phenotype possess proinflammatory functions. FACS results showed that in HFCFG-fed mouse model, the percentage of Ly6C^−^ MHC II^+^ macrophages increased by 54.6%, while CePA significantly reduced the number of macrophages to 32.7% in this subgroup. These results suggest that CePA can repress HFCFG feeding-induced maturation of inflammatory macrophages. Macrophages can be polarized in response to external environmental signals, and each type of polarized cell has different immunomodulatory effects. M1-type macrophages induce the expression of proinflammatory cytokines and present a stronger function of activating the immune response, which promotes liver damage in MASH [57]. Then, FACS was used to detect the infiltration of inflammatory cells in the liver tissue of HFCFG-fed mice. Moreover, CePA decreased the expression of the M1-like macrophage marker CD86 in liver tissues induced by HFCFG feeding but promoted the expression of the M2-like macrophage marker CD206. These results indicated that CePA could improve liver inflammation in the process of MASH by reducing the M1 polarization of macrophages (Fig. 7c).

Finally, we investigated the regulatory role of CePA in visceral adiposity and the systemic immune response. H&E staining and immunohistochemistry for F4/80 in HFCFG-fed and CePA-treated mice revealed crown-like structures, which are typical of macrophage infiltration. Indeed, combination of HFCFG feeding and CePA intervention resulted in an increase in macrophage filtration and a nearly 1.5-fold increase in F4/80 gene expression compared with CePA intervention. The results showed that CePA could reduce the diameter of white adipocytes and the infiltration of inflammatory cells in the epididymis (Fig. 7d and e). In conclusion, our findings demonstrated that CePA can effectively improve lipid deposition, steatosis, inflammatory cell infiltration, and liver fibrosis in both FA-stimulated hepatocytes and HFCFG-induced MASH models, suggesting that CePA can have a therapeutic effect on MASH. In addition, we also verified that CePA may play the above role by affecting the phosphorylation level of mTOR.

### Molecular mechanism of CePA protection against mTOR signaling and MASH progression

**Figure 8:**
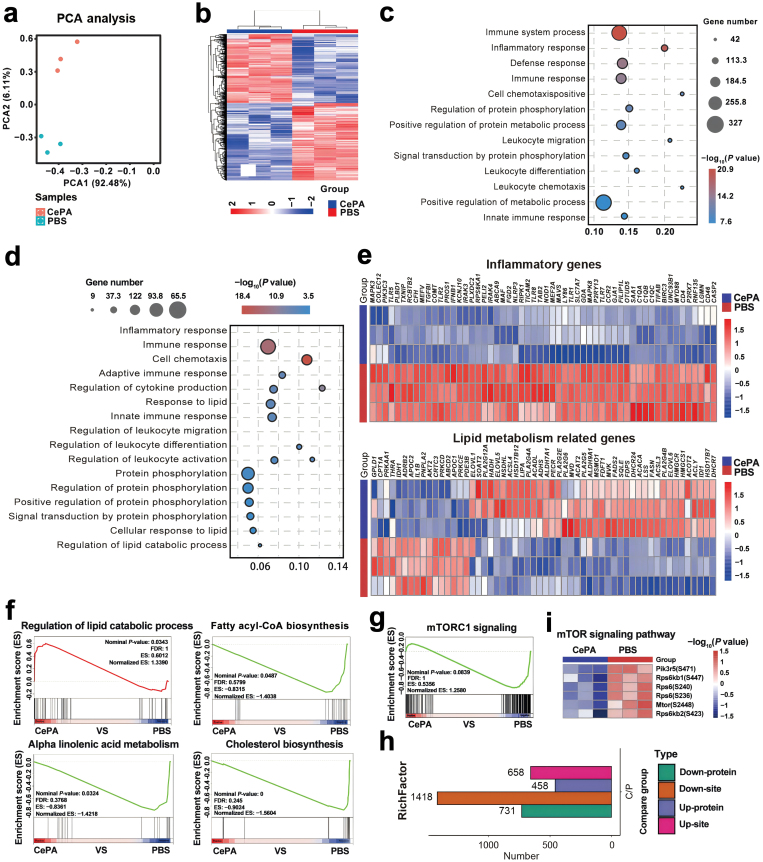
Bioinformatic analysis of the regulatory effect of CePA. (a) PCA after treatment with CePA. (b) Heatmap of differentially expressed genes from the PBS and CePA groups. (c) GO enrichment analysis to detect significantly differentially expressed genes related to inflammation and lipid metabolism. (d) KEGG pathway analysis to examine signaling pathways related to inflammation and lipid metabolism in downregulated genes. (e) Heatmap of differentially expressed genes of inflammation and lipid metabolism between the Ctrl and CePA groups based on the RNA-seq dataset. (f) GSEA to determine which lipid metabolism pathways are highly affected by CePA. (g) GSEA reveals that CePA significantly inhibited the mTORC1 signaling pathway. (h) Analysis of the expression-changed proteins and phosphorylation sites. (i) Heatmap of differentially expressed genes in the mTOR signaling pathway from the PBS and CePA groups.

The above results show that our CePA nanomaterial is effective in alleviating the symptoms of MASH, including lipid deposition, steatosis, and inflammation-related responses by affecting the phosphorylation of mTOR. To clarify the modulation mechanisms of the CePA-mTOR axis involved in MASH, RNA sequencing (RNA-seq) was performed by using liver tissue separated from PBS- and CePA-treated MASH model mice. Using our hepatic tissue for RNA-seq, we showed a principal components analysis (PCA) plot across hepatic samples. The PCA demonstrated the expected grouping among replicates within samples and sample groups spread across the two PCs. PCA showed clear clustering, suggesting that the sequencing results were more reliable and the liver tissue was more significantly altered after CePA treatment (Fig. 8a). After obtaining the lists of genes that were differentially expressed between the PBS and CePA groups, a heatmap was drawn, suggesting that 1249 genes were upregulated and 1119 genes were downregulated (Fig. 8b). A heatmap can provide a way to visually assess the results of clustering on the data, enabling us to observe the trends of gene expression under CePA treatment conditions. Next, we used clustering and identified our heatmap consisting of two groups, and we used these high-expression gene lists for functional enrichment analysis. Secondly, Gene Ontology (GO) functional analysis indicated that the immune system process was the most relevant of the differential pathways between PBS and CePA treatments, followed by the inflammatory response (Fig. 8c). A similar conclusion was also demonstrated in the Kyoto Encyclopedia of Genes and Genomes (KEGG) pathway clustering analysis (Fig. 8d). Therefore, we conducted in-depth screening of inflammatory genes and found that the expression levels of inflammatory genes, including Toll-like receptor (*TLR*) and mitogen-activated protein kinase (*MAPK*), were significantly reduced after CePA treatment. The results suggest that CePA can effectively alleviate mTOR signaling genes associated with inflammation in MASH. In addition, heatmap analysis of lipid metabolism-related genes was also conducted, and the results showed that CePA treatment significantly reduced genes, including *AKT*. A key part of RNA-seq analysis is the identification of individual genes or groups of genes that describe differences among groups. To allow the identification of the mechanisms of inflammation and metabolic changes, the expression of individual genes must be plotted to visualize and assess transcriptional changes at the gene level (Fig. 8e). At this level, we can analyze pathways of interest and assess efficacy. We further analyzed genes in gene set enrichment analysis (GSEA) to determine which lipid metabolism pathways were highly affected by CePA treatment and found that the lipid catabolic process gene set was strongly promoted, whereas fatty acyl-CoA biosynthesis, alpha-linolenic acid metabolism, and cholesterol biosynthesis pathways were significantly decreased (Fig. 8f). Further analysis screened the findings that CePA could inhibit the mTORC1 signaling pathway (Fig. 8g). mTOR is assembled into two distinct complexes, mTORC1 and mTORC2. Recent studies have shown that SREBP1 activation by Akt depends on mTORC1 but not mTORC2, and accumulating evidence suggests that mTOR-SREBP1 signaling is closely involved in *de novo* lipid synthesis in the liver. To better validate that CePA induces a series of molecular alterations dependent on the inhibition of mTOR phosphorylation levels, we performed mass spectrometry-based quantitative phosphoproteomic profiling to globally define the phosphorylation and dephosphorylation sites after CePA treatment. There were 731 downregulated and 458 upregulated phosphoproteins and 1418 downregulated and 658 upregulated phosphorylation sites (Fig. 8h). MASH pathogenesis is considered to be associated with the mTOR signaling pathway. Next, we screened and analyzed the mTOR signaling pathway separately. A heatmap showed decreased phosphorylation of mTOR signaling proteins, including mTOR (S2448), after CePA treatment (Fig. 8i).

## Conclusion

**Figure 9:**
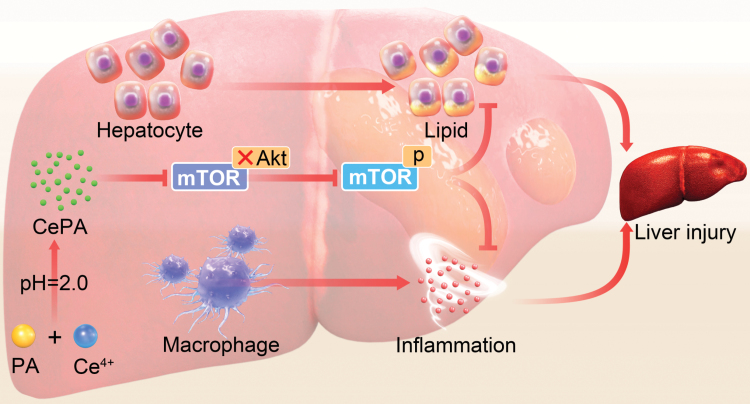
Synthetic procedures of CePA and mechanism of CePA targeting the mTOR signaling pathway. Cerium-CePA is demonstrated as an efficient nanomaterial to target the mTOR signaling pathway. After CePA enters the body, it reaches the liver tissues through the blood vessels. CePA could not only inhibit the binding of mTOR and Akt but also effectively inhibit the phosphorylation of mTOR and the inflammatory cell infiltration, improving liver tissue fibrosis in MASH.

In the present study, we demonstrated that targeted induction of mTOR regulation in the liver by CePA synergistically ameliorated obesity, inflammation, and MASH (Fig. 9). This study confirmed that CePA inhibited the inflammatory response and lipid metabolism and ameliorated liver injury in HFCFG-fed mice. Importantly, we constructed a CePA/mTOR/SREBP1 axis that regulates hepatic lipid metabolism and validated the inflammatory response modulation of MASH by CePA via mTOR. We comprehensively validated the targeting of CePA on mTOR at the *in vivo*, *ex vivo,* and bioinformatic levels, which provides a theoretical basis for the prevention and treatment of MASH. In future studies, we will deeply evaluate the regulatory mechanism of CePA and whether CePA can be involved in the regulation of liver cirrhosis and even hepatocellular carcinoma. Thus, the occupation inhibition strategy by targeting specific protein posttranslational modifications in liver injury is not limited to the regulation of phosphorylation levels. Further targeted modulation of multiple chemical modifications of liver cells will provide new ideas and translational prospects for more precise and flexible liver disease treatment. In conclusion, the regulation of MASH by CePA is closely related to mTOR. In our study, we identified that our CePA nanomaterial is effective in alleviating the symptoms of MASH, including lipid deposition, steatosis, and inflammation-related responses [58].

## Materials and methods

### Synthesis of CePA

Briefly, 5 mL of 50 mmol/L Ce(NH_4_)_2_(NO_3_)_6_ and 5 mL of PA (1%) were added to 40 mL of pure water and then stirred at 25°C for 6 h. After that, dry purified CePA was acquired by centrifugation washing and vacuum drying, which was stored at 4°C for further use.

### Animals and the MASH model

Eight-week-old wild-type C57BL/6 mice were obtained from Beijing Vital River Laboratory Animal Technology Co., Ltd. (Beijing, China) and maintained in a specific pathogen-free (SPF) facility. The mice in the NC group were fed normal chow. To induce MASH phenotypes and symptoms, mice were fed with an HFCFG diet (23.1 g fructose and 18.9 g glucose in 1 L water) for 16 weeks [59]. To evaluate the effect of CePA, mice were fed a high-fat diet supplemented with 1% CePA. During the feeding period, the fasting body weight was measured every week. All animal experiments were approved by the Animal Experiment Administration Committee of the Fourth Military Medical University to ensure ethical and humane treatment of the animals. Fasting blood glucose was measured after 16 weeks.

### Isolation of mouse liver

Macrophages in the liver were isolated from the livers of NC/CePA/HFCFG/HFCFG-CePA mice. After anesthetization with pentobarbital sodium (90 mg/kg body weight), the blood from the liver was flushed by injecting normal saline via a catheter in the left main cardiac chamber before collecting the livers of mice. The livers were then excised and minced, and liver bulk was cut into 1-mm pieces with scissors, followed by digestion at 37°C for 40 min shaking with collagenase IV and DNase I in Hank’s solution. Next, the media were passed through a 70-μm cell strainer and centrifuged at 350 *g* for 5 min to precipitate single cells. To isolate the nonparenchymal cells (NPCs), single-cell suspensions were centrifuged at low speed (50 *g*) for 4 min, and the supernatant was collected. The supernatant was then collected and processed for centrifugation at normal speed (1300 rpm) to pellet NPCs and then fractionated by 30% Percoll gradient centrifugation. Finally, macrophages were isolated by magnetic bead sorting (MojoSort™ Streptavidin Nanobeads). Centrifugation was repeated twice at 50 *g* to separate the hepatocytes from the NPC population. A further purification of the filtrate was performed in a 50% Percoll solution (GE Healthcare Life Sciences, #17-0891-01).

### Cell culture and stimulation

Bone marrow cells were isolated from femurs and tibias of 6- to 8-week-old C57BL/6 mice. After being strained with 70-µm strainers (Fisher Scientific) and lysing red blood cells with buffered 0.14 mol/L NH_4_Cl, bone marrow-derived macrophages (BMDMs) were induced by using 25 ng/mL murine macrophage-colony stimulating factor (M-CSF, SinoBio, Beijing, China) and cultured in Dulbecco’s modified Eagle’s medium (DMEM, Gibco, Waltham, MA, USA) with 10% fetal calf serum (FCS, Gibco) for 7 days. In several experiments, BMDMs were polarized into M1-polarized macrophages with LPS (10 ng/mL, Sigma) and IFN-γ (20 ng/mL, SinoBio) and treated with PBS, PA, CePA, FFA (OA:palmitic acid = 600 μmol:200 μmol), and CePA + FFA for 24 h. The human primary hepatocytes were all obtained from iXCells biotechnologies (Lot Number: 202715). The NH cell lines of mice (AML12) were all obtained from the American Type Culture Collection (ATCC, Manassas, VA) repository from 2015 to 2019. These cells were authenticated by both morphological profiling and short tandem repeat profiling and tested by PCR to exclude mycoplasma contamination. AML12 cells were treated with FA-free BSA or 30 mmol/L OA for 24 h before harvest.

### RNA extraction and reverse transcription-polymerase chain reaction (RT-PCR)

Total RNA was extracted separately from liver tissue and cells with TRIzol Reagent (15596-026; Invitrogen) and was then reverse transcribed into cDNA using a kit according to the manufacturer’s instructions. SYBR Green PCR Master Mix (Q311-02, ChamQ SYBR qPCR Master Mix, Vazyme) was used to perform RT-qPCR according to the manufacturer’s protocol. The relative mRNA expression levels of target genes were normalized to that of the housekeeping gene *β-actin*. The primers used for PCR were as follows:

*acc* forward: 5ʹ-ggaccactgcatggaatgttaa, *acc* reverse: 5ʹ-tgagtgactgccgaaacatctc;

*fasn* forward: 5ʹ-gcagtttcttgatgtggaacacagc, *fasn* reverse: 5ʹ-ttgtagtcagcacccaagtcctcg;

*srebp1* forward: 5ʹ-Taggtgtatttgctggcttggt, *srebp1* reverse: 5ʹ-agagatgactagggaactgtgtgt;

*acly* forward: 5ʹ-cagacgggcaaagaactcct, *acly* reverse: 5ʹ-tcaggagtgacccgagcata;

*cpt1α* forward: 5ʹ-agaggggaggacagagactgtacgc, *cpt1α* reverse: 5ʹ-caacctccatggctcagacagtacc;

*ppara* forward: 5ʹ-tcagggtaccactacggagt, *ppara* reverse: 5ʹ-cttggcattcttccaaagcg;

*sma* forward: 5ʹ-cagccccagtccctgaatt, *sma* reverse: 5ʹ-aagaggaagacagcacagctc;

*timp* forward: 5ʹ-gcatggacatttattctccactgt, *timp* reverse: 5ʹ-tctctaggagccccgatctg;

*il-1β* forward: 5ʹ-gagcaccttcttttccttcatctt, *il-1β* reverse: 5ʹ-tcacacaccagcaggttatcatc;

*il-6* forward: 5ʹ-caacgatgatgcacttgcaga, *il-6* reverse: 5ʹ-ctccaggtagctatggtactccaga;

*il-10* forward: 5ʹ-ccaagccttgtcggaaatga, *il-10* reverse: 5ʹ-gctagaagcatttgcggtgg;

*tgf-β* forward: 5ʹ-gcctgagtggctgtcttttg, *tgf-β* reverse: 5ʹ-ctgtattccgtctccttggttc;

*β-actin* forward: 5ʹ-tcatcactattggcaacgacg, *β-actin* reverse: 5ʹ-aacagtccgcctagaagcac.

### Histological analysis

Mouse liver tissues were fixed with 10% neutral formalin and embedded in paraffin. Embedded liver blocks were sliced into 5-μm-thick sections and stained with H&E (Hematoxylin, G1004, Servicebio, Wuhan, China; Eosin, BA-4024, Baso, Zhuhai, China), Masson (G1006, Servicebio, Wuhan, China), and Picrosirius Red (PSR) (26357-02, Head biotechnology, Beijing, China) to visualize the lipid accumulation pattern and liver fibrotic content. The NAS was derived based on the severity of hepatic steatosis (with a score of 0–3), lobular inflammation (with a score of 0–3), and hepatocellular ballooning (with a score of 0–2).

### Oil Red O staining

Frozen liver tissues that were embedded in Tissue-Tek OCT compound (4583, SAKURA, Torrance, CA) were stained with Oil Red O (O0625, Sigma, St. Louis, MO, USA) to evaluate lipid droplet accumulation. The cells were washed 2–3 times with PBS and fixed with paraformaldehyde at room temperature for 20 min. Then, they were washed 2–3 times with PBS and rinsed with 60% isopropanol solution for 20 s. Finally, the cells were stained with 60% filtered Oil Red O (O1391, Sigma Aldrich) at room temperature for 1 min. The histological features of the tissues were observed and imaged using a light microscope (ECLIPSE 80i. Nikon, Tokyo, Japan).

### Fluorescent immunohistochemistry

For immunofluorescence, the slides were incubated with anti-F4/80 primary antibody (dilution: 1:500, ab6640, Abcam, Southampton, UK), anti-iNOS (dilution: 1:200, ab49999, Abcam, Southampton, UK), and anti-ROS (dilution: 1:100, #3287, CST, Boston, USA) at 4°C overnight. Then, the sections were stained with a fluorophore-conjugated secondary antibody. The nuclei were stained with DAPI (dilution: 1:1000, ab285390, Abcam, Southampton, UK). Immunofluorescence images were obtained using a fluorescence microscope.

### Serum assay and hepatic lipid assay

The serum levels of ALT (Mouse ALT ELISA kit, ab282882, Abcam), AST (Mouse AST ELISA Kit, ab263882, Abcam), CHO (Cholesterol/Cholesteryl Ester Assay Kit, ab65359, Abcam), FAO (Fatty Acid Oxidation (FAO) Assay Kit, BR00001, AssayGenie), FFA (Free Fatty Acid Assay Kit, ab65341, Abcam), and blood glucose (CSB-E15775m, Cusabio) were measured according to the manufacturer’s instructions. The levels of liver and hepatocyte samples were measured using commercial kits.

### CoIP and western blot assay

Cells were lysed in IP lysis buffer (50 mmol/L Tris HCl, pH 7.4, 150 mmol/L NaCl, 1% NP-40 [Roche, 11,332,473,001], 0.5% sodium deoxycholate [Sigma, D6750], 0.1% SDS, and protease inhibitor cocktail [Roche, 4,693,116,001]). The lysates were immunoprecipitated with 1–3 µg of specific antibody and 25 µL A/G agarose beads (Sigma, 88,804) for 2 h at room temperature. The precipitates were washed three times with lysis buffer, and the immune complexes were boiled with loading buffer for 10 min before analysis by SDS-PAGE. For the western blotting assay, cells were lysed with RIPA buffer containing phenylmethanesulfonyl fluoride (PMSF) and a phosphatase inhibitor. After centrifugation at 12,000 *g* for 30 min, total protein was extracted, and the protein concentration was determined with a BCA kit. The protein samples mixed with loading buffer were subjected to 6% or 10% SDS-PAGE and transferred onto a PVDF membrane (IPVH00010; Millipore, Billerica, MA, USA). Membranes were blocked with 5% BSA (Beyotime Biotechnology, ST023) at room temperature for 1 h and then probed with indicated primary antibodies overnight at 4°C, followed by incubation with the appropriate HRP-conjugated secondary antibodies at room temperature for 2 h. After washing with TBST for three times, the blotted membranes were visualized with SuperSignal West Pico chemiluminescent substrate (Pierce Chemical, 34,580).

### OCR and ECAR analysis

OCR and ECAR were measured using a Seahorse XF24 Analyzer (Seahorse Bioscience). Briefly, seeding (4 × 10^4^ cells per well) was carried out in XFe24 Cell Culture Microplates. For OCR measurement, cells were covered with 500 µL assay medium (XF base medium (Seahorse, 102353), 1 mmol/L sodium pyruvate, 1 mmol/L L-glutamine, and 10 mmol/L glucose). Port injections were performed with 1 mmol/L oligomycin, 3 mmol/L FCCP, 0.5 mmol/L antimycin, and rotenone. For ECAR measurement, cells were covered with 500 µL assay medium (XF base medium, 1 mmol/L L-glutamine). Port injections were performed with 10 mmol/L glucose, 1 mmol/L oligomycin, and 50 mmol/L 2-deoxy-d-glucose (2-DG).

### Flow cytometry

The cells were stained with different antibodies. FACS analysis was performed according to routine protocols using a FACS AriaIII flow cytometer (BD Immunocytometry Systems). The data were analyzed using FlowJo vX.0.6 software (FlowJo, LLC, Ashland, OR, USA).

### RNA-seq and bioinformatic analysis

Total RNA was extracted and then used to construct cDNA libraries for profiling gene expression differences. A BGISEQ 500 instrument (MGI Tech Co., Ltd) was used for single-end sequencing of libraries. The reads were mapped to Ensembl mouse reference genomes by HISAT2 software (version 2.1.0). SAMtools (version 1.4) was used to generate a binary alignment map. Raw counts of genes were calculated by StringTie. To normalize the count matrix, DESeq2 (version 1.2.10) software was applied.

### GSEA

GSEA was implemented on the Java GSEA platform. For each KEGG biological pathway, the genes involved were defined as a gene set, and a ranked list and a "gene set" permutation were then generated. Gene sets with a *P* value of < 0.05 and a false discovery rate value of < 0.25 were considered statistically significant.

### Gene set variation analysis (GSVA)

GSVA was carried out to estimate samplewise KEGG pathway activity variation using the GSVA R package (version 1.32.0). For each KEGG pathway with at least 10 genes, the gene expression matrix was subjected to the calculation of the single-sample GSEA scores.

### Statistical analysis

All data are presented as the mean ± SD. The difference between two groups was tested by Student’s *t*-test. Comparisons among multiple groups were performed with one-way ANOVA. *P* values were specified as follows: ^*^P < 0.05; ^**^P < 0.01; ns, not significant.

## Supplementary Material

loae026_suppl_Supplementary_Materials

## Data Availability

All study data are included in the article and/or Supplementary Materials. Data are made available on request.
