# Correction to: Phytic acid-based nanomedicine against mTOR represses lipogenesis and immune response for metabolic dysfunction-associated steatohepatitis therapy

**DOI:** 10.1093/lifemeta/loaf043

**Published:** 2026-04-27

This is a correction to: Fenghua Xu, Shoujie Zhao, Yejing Zhu, Jun Zhu, Lingyang Kong, Huichen Li, Shouzheng Ma, Bo Wang, Yongquan Qu, Zhimin Tian, Junlong Zhao, Lei Liu, Phytic acid-based nanomedicine against mTOR represses lipogenesis and immune response for metabolic dysfunction-associated steatohepatitis therapy, *Life Metabolism*, Volume 3, Issue 6, December 2024, loae026, https://doi.org/10.1093/lifemeta/loae026.

In the published paper, there is a mistake regarding the order of the acknowledged grants in the PDF version. The acknowledgements should be:

“This work was supported by the National Key Research and Development Program of China (2021YFB3500904), the National Natural Science Foundation of China (82373409, 82173143, and 82173082), the Top Young Talents Project of “Special Support Program for High Level Talents” in Shaanxi Province (TZ0422, 2020−2025), and the 2023 program for training talented pilots upon graduation (LHRCYB23009). The authors also acknowledge the support from the Key Research and Development Program of Shaanxi (2024SF-YBXM-439), and the support from the ­Fundamental Research Funds for the Central Universities (D5000210635 and D5000210829).”

The error is outlined only in this notice to preserve the version of record.

