# Supplementary material for: Phytic acid-based nanomedicine against mTOR represses lipogenesis and immune response for metabolic dysfunction-associated steatohepatitis therapy
Source: Life Metab. 2024 Jun 18;3(6):loae026. doi: 10.1093/lifemeta/loae026 (PMC11748520; doi:10.1093/lifemeta/loae026)
Supplement: loae026_suppl_Supplementary_Materials [file loae026_suppl_Supplementary_Materials.docx]

**Phytic acid-based nanomedicine against mTOR represses lipogenesis and immune response for metabolic dysfunction-associated steatohepatitis therapy**

Fenghua Xu^1,2,‡^, Shoujie Zhao^3,‡^, Yejing Zhu^1,‡^, Jun Zhu^1,‡^, Lingyang Kong^2^, Huichen Li^1^, Shouzheng Ma^4^, Bo Wang^3^, Yongquan Qu^5,*^, Zhimin Tian^5,*^, Junlong Zhao^6,7,*^& Lei Liu^1,*^

^1^State Key Laboratory of Holistic Integrative Management of Gastrointestinal Cancers and Xijing Hospital of Digestive Diseases, Air Force Medical University, Xi’an, Shaanxi 710032, China

^2^MOE Key Laboratory of Modern Teaching Technology, Shaanxi Normal University, Xi’an, Shaanxi 710062, China

^3^Department of General Surgery, Tangdu Hospital, Air Force Medical University, Xi’an, Shaanxi 710038, China

^4^Department of Thoracic Surgery, Tangdu Hospital, Air Force Medical University, Xi’an, Shaanxi 710038, China

^5^Key Laboratory of Special Functional and Smart Polymer Materials of Ministry of Industry and Information Technology, School of Chemistry and Chemical Engineering, Northwestern Polytechnical University, Xi’an, Shaanxi 710072, China

^6^State Key Laboratory of Holistic Integrative Management of Gastrointestinal Cancers, Department of Medical Genetics and Developmental Biology, Air Force Medical University, Xi’an, Shaanxi 710032, China

^7^Department of Pediatrics, Tangdu Hospital, Air Force Medical University, Xi’an, Shaanxi 710000, China

^‡^These authors contributed equally to this work.

^*^**Corresponding authors.** Key Laboratory of Special Functional and Smart Polymer Materials of Ministry of Industry and Information Technology, School of Chemistry and Chemical Engineering, Northwestern Polytechnical University, Xi’an, Shaanxi 710072, China. E-mail: [yongquan@nwpu.edu.cn](mailto:yongquan@nwpu.edu.cn) (Y.Q.); [zhimintian@nwpu.edu.cn](mailto:zhimintian@nwpu.edu.cn) (Z.T.); State Key Laboratory of Holistic Integrative Management of Gastrointestinal Cancers, Department of Medical Genetics and Developmental Biology, Air Force Medical University, Xi’an, Shaanxi 710032, China. E-mail: [bio_junlongzhao@163.com](mailto:bio_junlongzhao@163.com) (J.Z.); State Key Laboratory of Holistic Integrative Management of Gastrointestinal Cancers and Xijing Hospital of Digestive Diseases, Air Force Medical University, Xi’an, Shaanxi 710032, China. E-mail: liulei84207@163.com

**
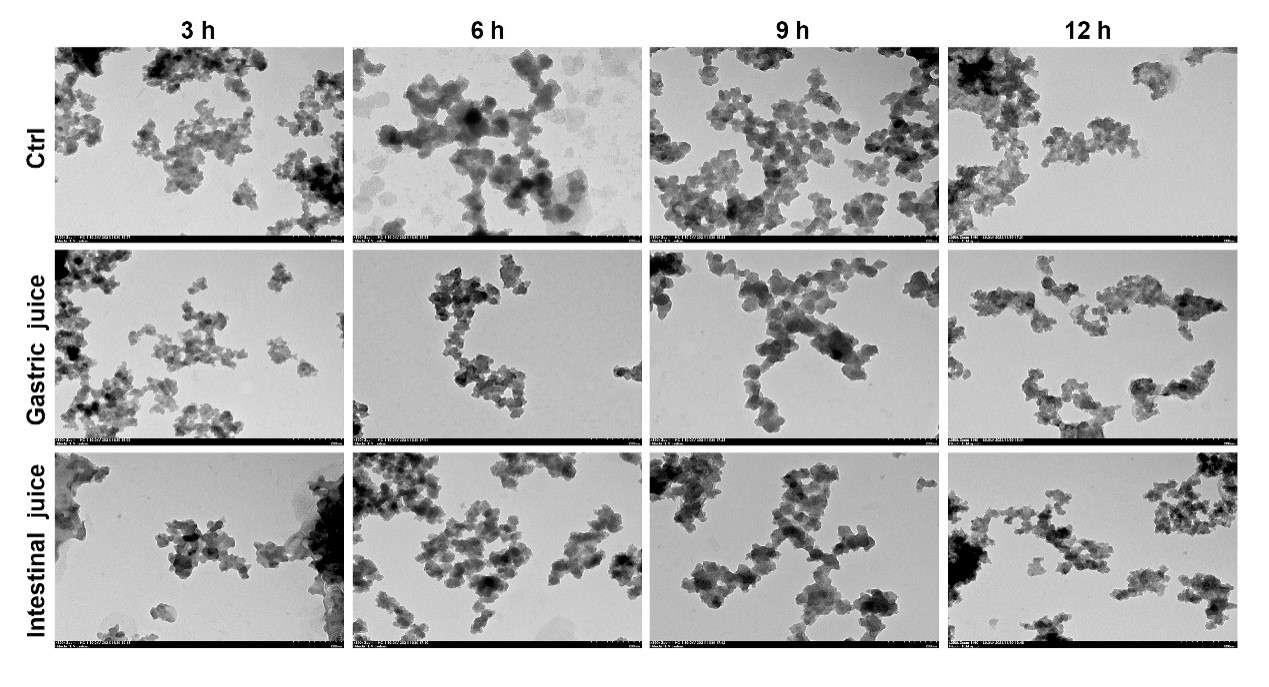
**

**Supplementary Figure S1** CePA can be digested and metabolized in the body. The figures show the morphological structure of CePA digested in the small intestine for 3 h, 6 h, 9 h, and 12 h by transmission electron microscopy. *n* = 3.

**
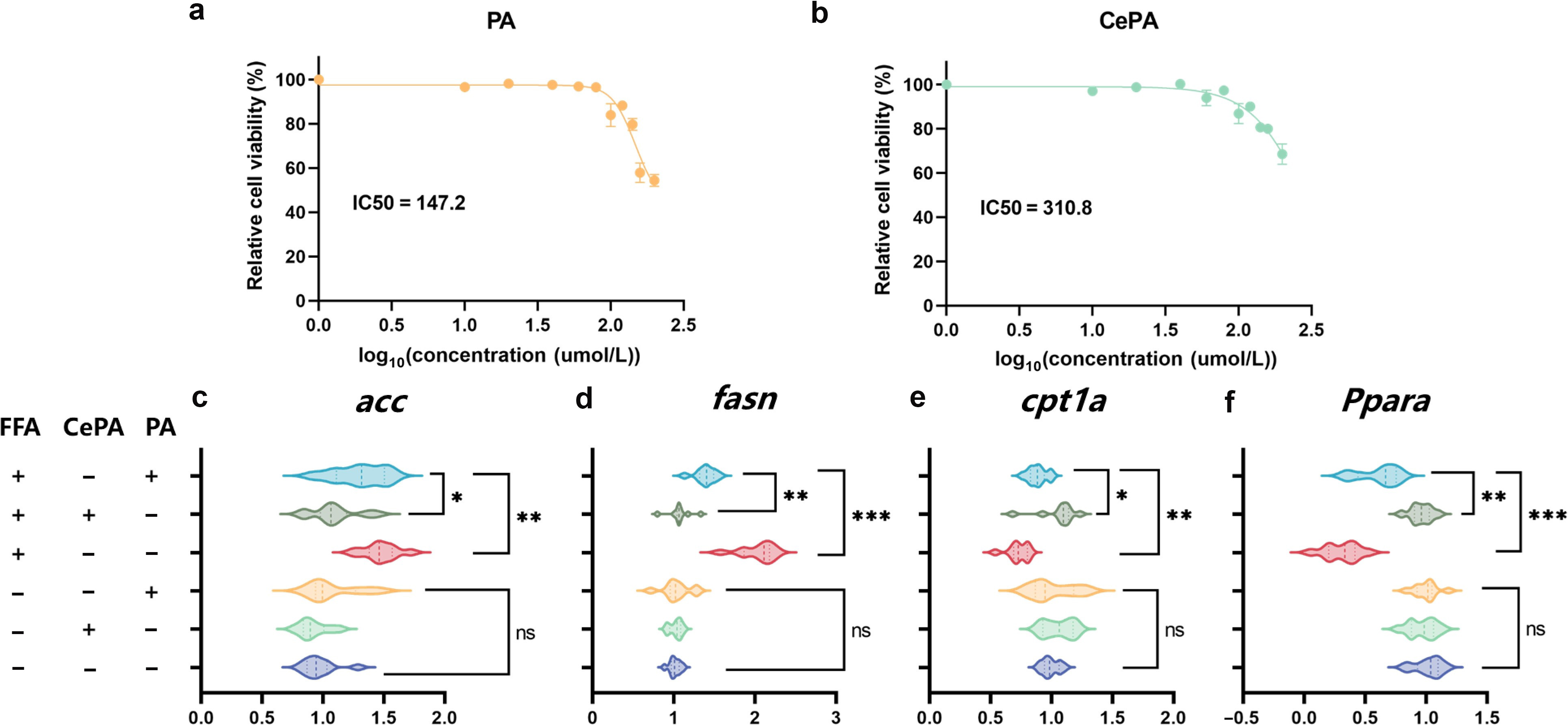
**

**Supplementary Figure S2** CePA is more stable and effective than PA. (a) Cell viability after PA treatment. (b) Cell viability after CePA treatment. (c) RT-PCR detection of mRNA levels for lipid metabolism-related genes (*acc*, *fasn*, *cpt1a*, and *Ppara*) in AML12 treated with or without CePA and PA after FFA stimulation for 24 h. *n* = 3. The data are expressed as the mean ± SEM; ^*^*P* < 0.05, ^**^*P* < 0.01, ^***^*P* < 0.001 vs. PBS.

**
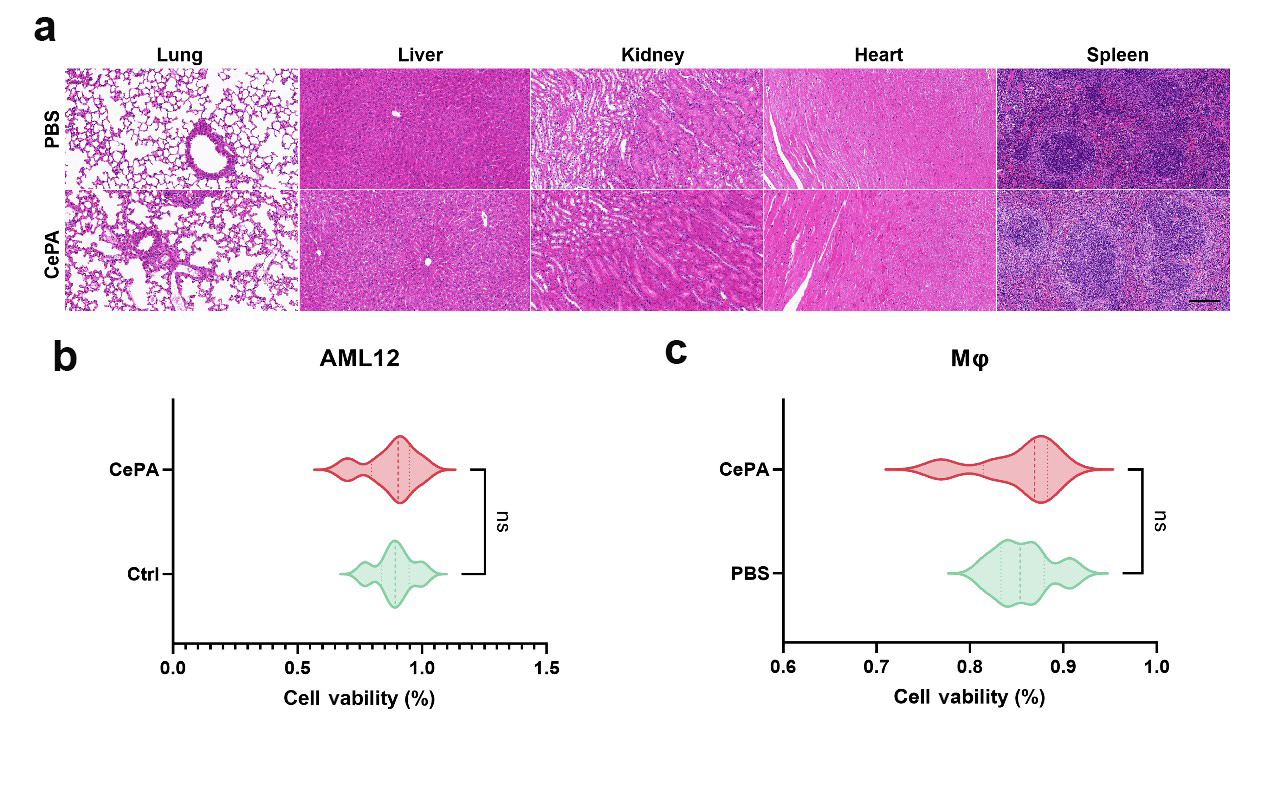
**

**Supplementary Figure S3** Biosafety of CePA. (a) H&E staining of mice in Ctrl, CePA, HFD, HFD + CePA groups. Scale bar, 100 u. (b) The AML12 and Mφ cell viability after CePA treatment. *n* = 3. The data are expressed as the mean ± SEM.
